# Prevalence and Associated Factors of Chronic Kidney Disease among Adult Hypertensive Patients at Northwest Amhara Referral Hospitals, Northwest Ethiopia, 2020

**DOI:** 10.1155/2021/5515832

**Published:** 2021-08-26

**Authors:** Anteneh Hunegnaw, Habtamu Sewunet Mekonnen, Masresha Asmare Techane, Chilot Desta Agegnehu

**Affiliations:** ^1^University of Gondar, Comprehensive Specialised Hospital, Gondar, Ethiopia; ^2^Department of Medical Nursing, School of Nursing, College of Medicine and Health Sciences, University of Gondar, Gondar, Ethiopia; ^3^Department of Pediatrics and Child Health Nursing, School of Nursing, College of Medicine and Health Sciences, University of Gondar, Gondar, Ethiopia; ^4^School of Nursing, College of Medicine and Health Sciences and Comprehensive Specialized Hospital, University of Gondar, Gondar, Ethiopia

## Abstract

**Background:**

Chronic kidney disease (CKD) is a progressive loss of the kidney function which leads to a decreased kidneys' ability to process waste in the blood and it affects the other important functions of the kidney. The disease has different stages that can alter the health status of individuals. During the early stages, patients may present with a normal or slight decrease in glomerular filtration rate (GFR) and albuminuria. Later, it progresses and leads to end-stage renal disease (ESRD) or kidney failure. Hypertension is considered as the major contributing risk factor of CKD.

**Objective:**

This study was aimed to assess the prevalence and associated factors of chronic kidney disease among adult hypertensive patients in referral hospitals of the Northwest Ethiopia.

**Methods:**

An institution-based cross-sectional study was conducted among 581 adult hypertensive patients in a chronic follow-up clinic in referral hospitals, Northwest Ethiopia, from July to August 2020. Systematic random sampling was used to select the study participants. Data were collected using the interviewer-administered questionnaire and participants medical records. Both bivariable and multiple logistic regression analyses were performed. Model fitness was assessed using a Hosmer—Lemeshow test.

**Result:**

The total prevalence of CKD among adult hypertensive patients was 17.6% (95% CI: 14.7–20.8). Diastolic blood pressure ≥90 mmHg (AOR = 8.65; 95% CI: 4.77–15.68), duration of hypertension ≥10 years (AOR = 8.81; 95% CI: 2.47–31.45), stage II HTN (AOR = 2.61; 95% CI: 1.04–6.50), comorbid disease (AOR = 7.0; 95% CI: 2.20–22.21), proteinuria (AOR = 4.59; 95% CI: 2.08–10.12), dyslipidemia (AOR = 3.40; 95% CI: 1.56–7.24), and serum creatinine ≥1 mg/dl (AOR = 8.88; 95% CI: 4.40–17.91) were associated with chronic kidney disease among adult hypertensive patients.

**Conclusion:**

In this study, the prevalence of CKD among hypertensive patients found was 17.6%. Regarding associated factors, dyslipidemia, proteinuria, comorbid disease, serum creatinine greater than 0.9 mg/dl, duration of hypertension greater than 10 years, and diastolic blood pressure greater than 90 mmHg are factors associated with the occurrence of chronic kidney disease among hypertensive patients.

## 1. Background

Chronic kidney disease (CKD) can be explained as a progressive loss of kidney function which results in decreased kidneys' ability to process waste and it affects the other important functions of the kidney [1]. The disease has different stages that can alter the health status of individuals. During the early stages, patients may present with a normal or slight decrease in glomerular filtration rate (GFR) and albuminuria. Later, it progresses and leads to end-stage renal disease (ESRD) or kidney failure [2–4]. Eventhough, the etiology of CKD still needs investigation [5]. Hypertension is considered as the major contributing risk factor of CKD [6, 7].

CKD usually gets worse over time though treatment has been shown to slow progression. If left untreated, it can progress to kidney failure and early cardiovascular disease. When the kidneys stop working, dialysis or kidney transplant is needed for survival. These would have negative impacts and burdens on patients, their families, and healthcare setups at large to the countries [8, 9].

Globally, the prevalence of CKD is significantly rising, and it is associated with major morbidity and mortality that demands special attention as one of the growing public health problems [10]. As it is evidenced by studies in the general population, the prevalence of CKD was 13.4% [10], while the pooled prevalence of CKD in patients with hypertensive was recorded higher as compared to the general population which is 24.7% [11]. Furthermore, as hypertension is the leading risk factor and causes for chronic kidney disease, the prevalence was higher compared with the general population [12].

CKD is associated with an increase in CVD and premature mortality, with detrimental societal and economic challenges [13, 14].

In 2017, CKD was the 12th leading cause of death worldwide and it resulted 1.2 million deaths, Besides, 7.6% of all CVD deaths (1.4 million) attributed to impaired kidney function. Deaths due to CKD or to CKD-attributable CVD accounted for 4.6% of all-cause mortality [15].

As the global burden of disease 2015 study, CKD was the leading cause of death and one of the fastest rising major causes of deaths in which overall mortality due to CKD has increased by 31.7% from 2005 to 2015 [16]. Moreover, in lower and middle-income countries, the prevalence is estimated to be 15% higher compared with high-income countries [17].

The prevalence of CKD among the general population in India, Korea, Pakistan, and Southern China were 17.2%, 13.7%, 12.5%, and 9.4%, respectively [6, 18, 19].

A systematic review in Africa continent indicates that the prevalence of CKD differs from 2% to 41%, and the prevalence among hypertensive patients is ranging from 13%–51%. This systematic review showed that the prevalence of CKD in Burundi, Mali, Nigeria, Egypt, Seychelles, Uganda, Congo, Kenya, Ghana, Cameroon, South Africa, Tanzania, Sudan, Senegal, and Morocco was 45.7%, 40.8%, 29.7%, 18%, 15.3%, 15.2%, 12.4%, 11.5%, 10.2%, 10%, 7.4%, 7%, 6.4%, 6.1%, and 2.9%, respectively [20].

A cross-sectional study conducted in Ethiopia at Tigray teaching hospital and Jimma University both indicate that the prevalence of CKD among hypertensive patients was 22.1% and 26%, respectively [21, 22].

As studies showed in the Glob, age, sex, educational status, residence, marital status, body mass index, comorbidity, type of antihypertensive medications, smoking, alcohol intake, and sedentary lifestyle were the associated factors with CKD [11, 18, 19, 21–28].

The World Health Organization (WHO) report, Ethiopia 2018, indicates that more than 39% of yearly deaths were recorded as a result of the noncommunicable disease including CKD and hypertension [12]. Additionally, CKD has a devastating effect on the nation's health sector and economy. However, very little is known about it in the study setting. Therefore, this study was aimed to assess the prevalence and associated factors of CKD among hypertensive patients.

## 2. Methods and Materials

### 2.1. Study Design and Period

An institution-based cross-sectional study was conducted from July to August/2020.

### 2.2. Study Area

The study was conducted in Northwest Ethiopia referral hospitals, namely, the University of Gondar Teaching Referral Hospital (UOGTRH), Felege Hiwot Referral Hospital (FHRH), and Debre Markos Referral Hospital (DMRH).

Each of them were estimated to serve for 5–7 million peoples in their catchment area, and UOGTRH has 1200 hypertensive patients, FHRH has 800 hypertensive patients, and DMRH has 600 hypertensive patients, which makes a total of 2600 hypertensive patients attending the chronic illness follow-up clinic in these catchment areas. UOGTRH is found in the Northern part of Amhara Region in Gondar town which has 9 nurses and 4 doctors who work in the chronic illness follow-up clinic regularly. FHRH is found in Bahirdar city, which is the capital city of Amhara Region, which has 8 nurses who work in the clinic regularly, and DMRH is again found at the east of Gojam in Amhara regional state, Northwest Ethiopia, which has 8 nurses who work in the clinic regularly.

### 2.3. Source and Study Populations

All hypertensive patients aged 18 years and above who had follow-up at the chronic illness clinic of referral hospitals in Northwest Ethiopia were the source population and those who attended during the data collection were the study population.

### 2.4. Inclusion Criteria

All adult hypertensive patients who had hypertension treatment follow-up at the three referral hospitals and who had medical records.

### 2.5. Exclusion Criteria

Pregnant women who were diagnosed with pregnancy-induced hypertension were excluded.

### 2.6. Sample Size Determination and Sampling Procedure

#### 2.6.1. Sample Size Determination

To get the adequate sample size, both the single population proportion formula and significantly associated factors from other study were computed (Table 1).

Accordingly, a single population proportion formula was calculated considering the following assumptions: 95% confidence interval (CI), 22.1% population proportion from the previous study [26], and 4% marginal error.

(1) $$\begin{array}{c} n=\frac{\left(Z\left(\alpha /2\right)\right)2*p\left(1-p\right)}{\left(d\right)2}, \end{array}$$

*n* represents the initial sample size; *Z α*/2 represents the standardized normal distribution value for the 95% CI = 1.96; *P* represents the proportion of chronic kidney disease (22.1%); *d* represents the margin of error 4%.

(2) $$\begin{array}{c} N=\frac{{\left(1.96\right)}^{2}\times 0.221\left(1-0.221\right)}{{\left(0.04\right)}^{2}}=413. \end{array}$$

By considering a 10% nonresponse rate, the sample size was 413 + 41 = 454.

By considering a 10% nonresponse rate, the final sample size was 528 + 53 = 581. Since the largest sample size (581) was found in associated factors, it was taken as the final sample size.

#### 2.6.2. Sampling Procedure

The total of 581 sample populations were proportionally allocated to each referral hospitals. About 268, 179, and 134 participants were selected in the University of Gondar Comprehensive Specialized Hospital, Felege Hiwot Referral Hospital, and Debre Markos Referral Hospital, respectively. Finally, study participants were selected using systematic random sampling as per their coming order. To avoid the recycling of data, special marks were put in participants' medical charts.

### 2.7. Variables of the Study

Dependent variable
  - Chronic kidney disease (yes/no)
Independent variables
  - Sociodemographic characteristics: age, sex, religion, educational status, occupation, income, marital status, residence, and family history of hypertension.
  - Clinical characteristics: BP status, baseline systolic BP, baseline diastolic BP, stage of hypertension, type of hypertension, type of drugs, duration of drugs, number of drugs, comorbidity, dyslipidemia, proteinuria, and obesity.
  - Lifestyle characteristics: smoking, alcohol use, and exercise.

### 2.8. Operational Definition

  Chronic kidney disease: CKD is defined and identified as when physicians diagnosed the patient as already having chronic kidney disease [29].
  Controlled blood pressure: BP < 140/90 mmHg in the last follow-up using a digital sphygmomanometer for adult hypertensive clients without diabetes mellitus and chronic kidney disease and blood pressure <130/80 mmHg using a digital sphygmomanometer for adult hypertensive clients with diabetes mellitus and chronic kidney disease in the last follow-up record [29].
  BMI (body mass index): A measure of the person's weight in kilogram divided by his height in meter square; based on this calculation, bodyweight was classified as underweight for a patient with less than 18.5 kg/m^2^, normal weight 18.5–24.9 kg/m^2^, overweight 25–29.9 kg/m^2^, and obese for patients with BMI ≥ 30 kg/m^2^ [2]
  Proteinuria: according to the laboratory result, if a patient had plus one and above, we classify as having proteinuria in the last laboratory result [29].
  Hypertension: it was defined as those who had a documented diagnosis of hypertension (i.e., BP ≥ 140/90 mmHg) or those on antihypertensive agents. Target control BP was defined as BP < 140/90 mmHg for nondiabetic and <130/80 mmHg for diabetic patients [30].
  Estimated GFR: this is a test that is used to assess how well kidneys are working. The test estimates the volume of blood that is filtered by the kidneys over a given period. It can be measured by using readily available equations and formulas like Cockcroft–Gault. Cockcroft–Gault formula (normalized for the body surface area (BSA)): (140 − age (years)) × weight (kg) × (0.86, if female) × 1.73/72 × serum creatinine (mg/dL) × BSA (m^2^). In this case, BSA will be calculated by using the Mosteller formula, that is, BSA is equal to $\sqrt{W\times H}$ divided by 60, while weight is measured in kilogram and height in centimeter [31].
  Alcohol exposure: participant; intake of alcohol, according to the patient's record of taking any alcoholic drinks will be considered as alcohol consumer [21].
  Smoking: the patient's response/record of who smokes or ever smoke [32].
  Physical exercise: the patients say they do perform the daily active exercise for about 30 minutes a day [28].
  Comorbidity: it is the presence of any health conditions other than hypertension.

### 2.9. Data Collection Tool and Procedure

A standardized tool was used for data extraction. The tool contains sociodemographic and baseline clinical and treatment-related variables. In addition to face-to-face interview, forms were used for laboratory request and follow-up records of hypertension, and patient cards were reviewed.

Two days training was given for data collectors regarding each description of the tool and the way of data collection from patients and their medical records using the extraction tool. For the sake of appropriate data collection, four nurses working in a chronic clinic or at least who have previous experience in an outpatient chronic clinic participated in data collection; one health officer has participated as a supervisor. Then, data were collected from the patient and their medical charts by using a prepared extraction tool. In this way, all patients who fulfill the inclusion criteria were interviewed, and data extraction was completed.

### 2.10. Data Quality Control

To maintain data quality, an appropriate and standardized extraction tool was used. The data extraction tool was pretested for consistency and completeness of data items on 5% [25] of patient charts at the same hospital. Quality was also maintained by using experienced nurses in the chronic clinic. Even training was given to data collectors for one day before the data collection on the objective of the study and how to extract data for this study purpose using the data extraction format. Each component of the tool was discussed clearly for data collectors. The data extraction process was monitored closely by the supervisor throughout the data collection period. Data clerks also assist data collectors by identifying the charts. After filling each tool, completeness was checked, and the correction was made before the chart returned.

### 2.11. Data Processing and Analysis

Data were entered into Epi-Info version 7.2.2.6 and then transferred to SPSS version 23 for analysis. Summary statistics were used to look for any missing, and logistic regression was also used to determine crude and adjusted odds ratio (OR). Bivariate analysis was carried out to assess an association between the dependent and all the independent variables, and variables with a *p* value <0.2 were further entered into the multivariable logistic regression model to control the possible effect of confounders. Model fitness was assessed using the Hosmer–Lemeshow test (0.223). Odds ratios with 95% confidence interval at *p* value <0.05, cleaning, and analysis were computed to determine the level of significance.

## 3. Results

### 3.1. Sociodemographic Characteristics of Participants

In this study, 581 patients were included with a response rate of 100%. Of these, 55.1% were females, the median and standard deviation of the age was 54.71 and 11.397, respectively, 476 (81.9%) were married, 120 (20.7%) had no formal education, 429 (73.8%) were urban inhabitants, 497 (85.5%) were orthodox followers, and 140 (24.1%) were government employee (Table 2).

### 3.2. Clinical Related Characteristics

Of 581 study subjects, 64 (11%) and 54 (9.3%) of them have greater than and equal to 140 mmHg for systolic and 90 mmHg for diastolic blood pressure, and 429 (73.8%) of them have a BMI of 18.5–24.9. Also, 288 (49.6%) of them are in the second stage of hypertension, 221 (38%) of them have the comorbid disease, and 394 (67.8%) of patients use calcium channel blockers for the treatment of hypertension (Table 3).

### 3.3. Lifestyle Factors

Among the 581 study subjects, 119 (20.5%) of them are alcohol users and only 28 (4.8%) of them are doing exercise on regular basis (Table 4).

#### 3.3.1. Prevalence of Chronic Kidney Disease among Adult Hypertensive Patients

The total prevalence of CKD among hypertensive patients was 17.6% (95% CI: 14.7–20.8).

#### 3.3.2. Associated Factors of Chronic Kidney Disease among Adult Hypertensive Patients

In bivariable analysis, age less than 60 years, systolic blood pressure greater than 140 mmHg, diastolic blood pressure greater than 90 mmHg, stage II and III hypertension, duration of hypertension greater than 10 years, comorbid disease, drugs other than hypertension, proteinuria, dyslipidemia, serum creatinine, body swelling, beta-blocker, diuretics, and vasodilators, smoking, alcohol use, and exercise were associated with chronic kidney disease at *p* value <0.2.

However, in multivariable analysis, DBP ≥90 mmHg, duration of hypertension ≥10 years, stage II HTN, proteinuria, dyslipidemia, and serum creatinine ≥0.9 mg/dl were associated with chronic kidney disease among hypertensive patients at *p* value <0.05.

The odds of CKD were 8.65 times (AOR = 8.65, 95% CI: 4.77–15.68) higher among those who had diastolic blood pressure greater than 90 mmHg as compared to their counter parts.

Patients with duration of hypertension ≥10 years were (AOR = 8.81; 95% CI 2.47–31.45) more likely to develop CKD as compared to patients with duration of hypertension <10 years.

Being stage II HTN patients was 2.61 (AOR = 2.61; 95% CI: 1.04–6.50) times higher among those patients at stage I HTN.

The odds of having CKD was 4.59 times (AOR = 4.59; 95% CI: 2.08–10.12) higher among proteinuria as compared to those patients free from proteinuria. Patients with the comorbid disease were 7 times (AOR = 7; 95% CI: 2.2–22.2) more likely to develop CKD as compared to their counter parts. Regarding to serum creatinine, patients who had creatinine greater than 1 mg/dl had 8.9 times (AOR = 8.9; 95% CI: 4.40–17.91) higher odds of CKD as compared to serum creatinine less than 1 mg/dl. Patients with dyslipidemia had 3.5 times (AOR = 3.40; 95% CI: 1.56–7.24)) higher odds of CKD as compared to patients with no dyslipidemia (Table 5).

## 4. Discussion

This study was determined the prevalence of CKD among adult hypertensive patients attending the chronic illness follow-up clinic at Northwest Ethiopia referral hospitals and identified associated factors with CKD among adult hypertensive patients.

In the current study, the prevalence of CKD among adult hypertensive patients was 17.6% (95% CI: 14.7–20.8%). This result was in line with the studies conducted in Jimma (26%) [22] and Addis Ababa Police Hospital (14.6%) [11].

However, the finding of this study was lower than the findings of studies conducted in Ghana (46.9%) [33], Nigeria (38.5%) [20], Burkina Faso (50.8%) [34], South China (23%) [35], Ethiopia (22%) [11], and South Africa (45.5%) [36]. This is might be due to the difference in a study subject, study setting, sample size difference, and outcome ascertainment difference. In the previous study conducted in Ghana, the included study participants were aged 16 years and above; whereas, in this study, the included study participants were aged 18 years and above. On the other hand, from the previous study, the settings were polyclinic; whereas, in this study, the study settings are referral hospitals.

This result was higher than studies conducted in Pakistan (12%) [30]. This difference might be due to differences in a study subject, sample size difference, the difference in the study setting, outcome ascertainment difference, and the contextual difference between the two countries.

According to this study, long duration of hypertension was associated with chronic kidney disease among adult hypertensive patients, which showed that the duration of hypertension greater than 10 years was almost nine times more likely to develop chronic kidney disease as compared to their counterpart. This finding was in agreement with the studies conducted in Ethiopia [11], Jimma [11], and South China [22]. This might be due to persistent increase of blood pressure predispose hypertensive patients to renal vascular nephropathy, which gradually leads to the decrement of glomerular filtration rate and allowing sufficient time to develop other renal diseases; in other way, nephrons in the kidney are supplied with a dense network of blood vessels, and the high volume of blood flow through them; overtime uncontrolled high blood pressure can cause arteries around the kidney narrow, weaken, or harden. These damaged arteries are not able to deliver enough blood to the kidney tissue [29].

In this study, presence of comorbid (diabetes and CHF) also associated with CKD among adult hypertensive patients showed that patients with the comorbid disease were 7 times more likely to develop chronic kidney disease as compared to their counterpart. This finding was similar to a study conducted in South Asia [30] and South China [33]. This might be due to diabetes if the patient produces little or no insulin, or if the body cannot use the insulin, the sugar remains in the bloodstream instead of going into the cells. Over time, high levels of sugar in the blood damage tiny blood vessels throughout the body including the filter of the kidney. As more damage occurs to the kidney, more fluid and waste remain in the bloodstream instead of being removed, and in patients with cardiac problems, cardiac output may be low in the glomerular filtration membrane [13].

This study also identified elevated diastolic blood pressure as a significant factor for CKD among adult hypertensive patients, which showed that patients with diastolic blood pressure greater than 90 mmHg were 8.65 times more likely to develop chronic kidney disease as compared to their counterpart. This might be due to patients with increased diastolic blood pressure can cause arteries around the kidney to narrow, weaken, or harden and damages filtration [17].

Based on this study, finding dyslipidemia was associated with CKD among adult hypertensive patients, which showed that patients with dyslipidemia were nearly three and half times more likely to develop chronic kidney disease as compared to their counterpart. This result is similar to a study conducted in Iran [37], Ethiopia [11], and South China [38]. This might be due to dyslipidemia may lace the patients for cardiovascular disease, and besides, it causes cholesterol plaque that can lead to clogging of renal arteries and cuff off blood flow to the kidneys resulting in the loss of kidney function, and also, triglyceride is more associated with cardiovascular risk factors when levels are too high [28].

According to this study, serum creatinine greater than 1 mg/dl was associated with chronic kidney disease among hypertensive patients, which showed that patients with serum creatinine greater than 1 mg/dl were 9 times more likely to develop chronic kidney disease among adult hypertensive patients as compared to their counterparts. This result is similar to a study conducted in India [35]. This might be due to as serum creatinine increase. It obstructs the epithelial cells and causes necrotic debris and back leak of filtrate through injured tubular epithelial cells, causing further reduction of the glomerular filtration rate [39].

According to this study, proteinuria was associated with chronic kidney disease among adult hypertensive patients, which showed that patients with proteinuria were 4.59 times more likely to develop chronic kidney disease as compared to their counterparts. This finding is similar to a study conducted in Ethiopia [21, 27].

According to this study, stage II HTN was associated with chronic kidney disease, which showed that patients with proteinuria were 2.61 times more likely to develop chronic kidney disease as compared to their counterparts. This finding is similar to a study conducted in India [35]. This might be due to the atherosclerotic, hypertension-related vascular lesions in the kidney primarily affecting the preglomerular arterioles, resulting in ischemic changes in the glomeruli and postglomerular structures. Glomerular injury may also be a consequence of direct damage to the glomerular capillaries due to glomerular hyperperfusion [30]. Since self-reporting was used to this method, it has the disadvantages of recall bias.

The limitation of this study was secondary data, as we could not get potential predictors. It shares also the limitation of the cross-sectional study design.

## 5. Conclusion

In this study, the prevalence of CKD among hypertensive patients was found to be 17.6%. Besides dyslipidemia, proteinuria, comorbid disease, serum creatinine greater than 1 mg/dl, duration of hypertension greater than 10 years, and diastolic blood pressure greater than 90 mmHg are factors associated with the occurrence of chronic kidney disease among hypertensive patients.

## Figures and Tables

**Table 1:** Sample size calculation using associated factors.

No.	Associated factors	Assumption	Number
1	BMI	OR = 1.38 [29], % = 32%, *p*=80, *R* = 1	528
2	Body swelling	OR = 3.01 [29], % = 32%, *p*=80, *R* = 1	122
3	Uncontrolled BP	OR = 2.92 [29], % = 25, *p*=80, *R* = 1	284

**Table 2:** Sociodemographic characteristics of hypertensive patients attending at the chronic follow-up clinic at Northwest Ethiopia referral hospitals, 2020.

Variable	Category	Frequency	Percentage
Sex	Male	261	44.9
	Female	320	55.1

Age	<60 years	394	67.8
	≥60 years	187	32.2

Marital status	Single	18	3.1
	Married	476	81.9
	Divorced	28	4.8
	Widowed	59	10.2

Religion	Orthodox	497	85.5
	Muslim	53	9.1
	Protestant	31	5.3

Level of education	No formal education	120	20.7
	Primary	124	21.3
	Secondary	164	28.2
	Diploma and above	173	29.8

Occupation	Government employee	140	24.1
	Farmer	66	11.4
	Private	92	15.8
	Merchant	45	7.7
	Housewife	159	27.4
	Others	79	13.6

Residence	Urban	429	73.8
	Rural	152	26.2

^∗^Others, retired and not yet working.

**Table 3:** Clinical factors of hypertensive patients attending at the chronic follow-up clinic at Northwest Ethiopia referral hospitals.

Factors	Category	Frequency	Percentage
DBP	<90	527	90.7
	≥90	54	9.3

SBP	<140	517	89.0
	≥140	64	11.0

Duration of HTN (years)	<10	550	94.7
	≥10	31	5.3

BMI	<18.5	15	2.6
	18.5–24.9	429	73.8
	25–29.9	94	16.2
	>30	43	7.4

Stage of HTN	Stage I	262	45.1
	Stage II	288	49.6
	Stage III	31	5.3

Comorbid disease	No	360	62.0
	Yes	221	38.0

Family Hx of HTN	No	541	93.1
	Yes	40	6.9

Drugs other than anti-HTN Rx	No	373	64.2
	Yes	208	35.8

Proteinuria	No	488	84.0
	Yes	93	16.0

Dyslipidemia	No	436	75.0
	Yes	145	25.0

Body swelling	No	522	89.8
	Yes	59	10.2

ACE inhibitor	No	297	51.1
	Yes	284	48.9

Beta-blocker	No	525	90.4
	Yes	56	9.6

Diuretics	No	329	56.6
	Yes	252	43.4

Calcium channel blocker	No	187	32.2
	Yes	394	67.8

Vasodilator	No	462	79.5
	Yes	119	20.5

**Table 4:** Lifestyle factors of hypertensive patients attending at chronic follow-up clinic at Northwest Ethiopia referral hospitals.

Factors	Category	Frequency	Percentage
Smoking habit	No	563	96.9
	Yes	18	3.1

Daily exercise	No	553	95.2
	Yes	28	4.8

Alcohol user	No	462	79.5
	Yes	119	20.5

**Table 5:** Bivariate and multivariate analyses of hypertensive patients attending at the chronic follow-up clinic at Northwest Ethiopia referral hospitals.

Variables	CKD		COR	AOR
	Yes	No		
SBP				
<140 mmHg	65	452	1	1
≥140 mmHg	37	27	9.529 (5.44, 16.68)	1.72 (0.57, 5.21)

DBP				
<90 mmHg	71	456	1	1
≥90 mmHg	31	23	8.656 (4.77, 15.68)	8.65 (4.77, 15.68)

Stage of HTN				
Stage I	9	253	1	1
Stage II	82	206	11.19 (5.48, 22.81)	2.61 (1.04, 6.50)
Stage III	11	20	15.46 (5.73, 42.67)	1.18 (0.26, 5.39)

Duration of hypertension				
<10 years	86	464	1	1
≥10 years	16	15	5.75 (2.74, 12.07)	8.81 (2.47, 31.45)

Comorbid disease				
No	91	130	1	1
Yes	11	349	22.20 (11.51, 42.8)	7.0 (2.20, 22.21)

Drugs other than HTN				
No	26	347	1	
Yes	76	132	7.64 (4.71, 12.52)	0.39 (0.14, 1.13)

Proteinuria				
No	43	445	1	1
Yes	59	34	17.95 (10.61, 30.37)	4.59 (2.08, 10.12)

Dyslipidemia				
No	27	409	1	1
Yes	75	70	16.23 (9.76, 26.96)	3.40 (1.56, 7.24)

Serum creatinine				
<1 mg/dl	29	434	1	1
>1 mg/dl	73	45	24.27 (14.31, 34.18)	8.88 (4.40, 17.91)

## Data Availability

The data used to support the findings of this study are included within the article, and the dataset analyzed is available from the corresponding author upon request.

## Abbreviations

BMI: Body mass index
BP: Blood pressure
CKD: Chronic kidney disease
CVD: Cardio vascular disease
DM: Diabetes mellitus
DMRH: Debre Markos referral hospital
FHRH: Felege Hiwot referral hospital
HIV/AIDS: Human immune virus/acquired immune deficiency syndrome
NSAIDs: Nonsteroidal anti-inflammatory drugs
UOGTRH: University of Gondar teaching referral hospital.
